# A Systematic Review and Meta-Analysis of Circulating Biomarkers Associated with Failure of Arteriovenous Fistulae for Haemodialysis

**DOI:** 10.1371/journal.pone.0159963

**Published:** 2016-07-26

**Authors:** Susan K. Morton, Alexander J. Rodríguez, Dylan R. Morris, Abhishta P. Bhandari, Joseph V. Moxon, Jonathan Golledge

**Affiliations:** 1 The Vascular Biology Unit, Queensland Research Centre for Peripheral Vascular Disease, College of Medicine and Dentistry, James Cook University, Townsville, Australia; 2 Bone and Muscle Health Research Group, Department of Medicine, Monash University, Melbourne, Australia; 3 Department of Vascular and Endovascular Surgery, The Townsville Hospital, Townsville, Australia; Sao Paulo State University, BRAZIL

## Abstract

**Background:**

Arteriovenous fistula (AVF) failure is a significant cause of morbidity and expense in patients on maintenance haemodialysis (HD). Circulating biomarkers could be valuable in detecting patients at risk of AVF failure and may identify targets to improve AVF outcome. Currently there is little consensus on the relationship between circulating biomarkers and AVF failure. The aim of this systematic review was to identify circulating biomarkers associated with AVF failure.

**Methods:**

Studies evaluating the association between circulating biomarkers and the presence or risk of AVF failure were systematically identified from the MEDLINE, EMBASE and Cochrane Library databases. No restrictions on the type of study were imposed. Concentrations of circulating biomarkers of routine HD patients with and without AVF failure were recorded and meta-analyses were performed on biomarkers that were assessed in three or more studies with a composite population of at least 100 participants. Biomarker concentrations were synthesized into inverse-variance random-effects models to calculate standardized mean differences (SMD) and 95% confidence intervals (CI).

**Results:**

Thirteen studies comprising a combined population of 1512 participants were included after screening 2835 unique abstracts. These studies collectively investigated 48 biomarkers, predominantly circulating molecules which were assessed as part of routine clinical care. Meta-analysis was performed on twelve eligible biomarkers. No significant association between any of the assessed biomarkers and AVF failure was observed.

**Conclusion:**

This paper is the first systematic review of biomarkers associated with AVF failure. Our results suggest that blood markers currently assessed do not identify an at-risk AVF. Further, rigorously designed studies assessing biological plausible biomarkers are needed to clarify whether assessment of circulating markers can be of any clinical value. PROSPERO registration number CRD42016033845.

## Introduction

A surgically created arteriovenous fistula (AVF) is the preferred form of long term vascular access (VA) for use in haemodialysis (HD) therapy. Recent statistics from the USA show that in 2015 66% of routine HD patients used an AVF for VA, and this proportion is predicted to increase in line with the national ‘Fistula First Catheter Last’ initiative [1]. Nevertheless, AVF failure resulting from complications such as venous stenosis and thrombosis remain a major cause of hospitalization and morbidity within the HD population [2]. The Dialysis Outcomes Quality Initiative (DOQI) has reported that primary AVF failure is approximately 15% after one year and 25% after two years [3]. Recent data suggest that AVF survival has not significantly improved more than a decade after standardised VA guidelines were introduced [4].

Currently AVFs are monitored using duplex ultrasound (US) to assess blood flow and identify flow disturbances in the AVF or adjacent vessels. While routine screening may improve AVF survival rates by allowing early identification and remediation of at-risk fistulae, US-based screening programs are time and labour intensive and rely on specialist equipment which is often unavailable at regional centres [5]. In contrast, screening programs based on the detection of blood-borne markers (biomarkers) could provide a more cost effective means to identify patients at risk of AVF failure.

Few studies have assessed the relationship between biomarkers and AVF failure, and discrepancy exists between the conclusions reported. To date, there has been no systematic evaluation of the available literature to clarify the reported association between circulating biomarkers and AVF failure. Accordingly, we performed a systematic review and meta-analysis of publicly available literature to examine the association of circulating biomarkers with AVF failure.

## Methods

### Search protocol and study focus

We performed a systematic literature review of published work in accordance with the MOOSE guidelines [6]. This review was registered in the PROSPERO International Registry, registration number CRD42016033845. We sought studies that investigated the association of at least one circulating biomarker with an AVF outcome (such as thrombosis, stenosis or failure) in patients receiving regular HD. We predominantly sought literature from the online MEDLINE (January 1966 to December 2015), EMBASE (January 1980 to December 2015) and the Cochrane Library databases as well as scanning reference lists of studies captured in the literature search. In performing literature searches, we applied the search terms “AVF” AND “vascular access”, as well as one of the following title/abstract phrases: “biomarker”, “concentration”, “function”, “dysfunction”, “maturation”, “patency”, “failure”, “survival”, “thrombo*”, “steno*”, “factor”, “predict*”, “serum”, “plasma”, “circulating”, “risk factor” and “blood” with no language restriction (See S1 File for details). Titles and abstracts of identified searches were screened and if the suitability of the article was uncertain, the full text was assessed. We considered a native AVF to mean the anastomosis of an artery and vein; and graft to mean the surgical placement of a loop or bypass (either from autologous tissue or synthetic material) to join an artery and vein. AVF failure was defined as complications in the VA which prevented successful HD, arising from events such as AVF stenosis or thrombosis.

### Study eligibility

Studies were deemed eligible if i) the patient population investigated were using, or were to receive a native AVF for HD; ii) the study assessed and reported the association of circulating biomarker(s) with the presence or risk of AVF failure; iii) cases were patients with a malfunctioning AVF from any reason (defined as AVF failure) and controls were patients whose AVF remained functioning and able to be used for HD (defined as a patent AVF); iv) specific details of the timing of blood collection relative to AVF failure were provided, and v) the full manuscript was in English. Specific exclusion criteria included i) animal model studies; ii) studies investigating non-surgically created AVFs; iii) non-HD related AVFs; and iv) studies evaluating multiple types of VA without providing AVF-specific results.

### Quality assessment, data extraction and biomarker selection for meta-analysis

Data extraction was performed using a standardised data extraction form (S2 File). The following clinical data were extracted from all studies: 1) General patient characteristics (e.g. age, sex and smoking); 2) Definitions of case and control groups; 3) Definitions of patent or failed AVF; 4) Timing of blood sampling relative to AVF failure, and blood medium assessed; 5) Outcome measures; 6) Methods of biomarker quantification; 7) Statistical analyses performed, including reported concentrations, effect estimates, variability, and p-values. The type of study design was also recorded (e.g. cohort or case control). Each study was assessed using a modified version of the Ottawa-Newcastle tool to assess the risk of bias. The assessment tools and subsequent results are provided within the supplementary material (S3 and S4 Files for cohort and case control studies, respectively). Risk of bias was classified very low, low, medium, high or very high, depending on the assessment outcome (see S3 and S4 Files for specific details regarding cohort and case control studies, respectively).

Biomarkers which were assessed by ≥3 independent studies in a composite population of ≥100 patients were included in a meta-analysis. Results of the meta-analysis are presented as mean and standard deviation (SD). Where studies presented data as mean and standard error of the mean (SEM), standard deviation was recalculated using the following equation: *SD* = *SEM* × √*n*, where n = population size.

### Meta-analysis

Biomarker concentrations were compared between patients with (cases), and without (controls) AVF failure. An inverse-variance random-effects model was applied to determine the standardised mean difference (SMD) and 95% confidence intervals (95% CI) of biomarker concentrations between case and control groups. Inter-study heterogeneity was determined using the I^2^ index and its associated p-value, as detailed by detailed by Higgins *et al* [7]. All statistical analyses were performed using RevMan v5.3 software (The Nordic Cochrane Centre, The Cochrane Collaboration, 2014) and GraphPad Prism v6.05 software. For all reported comparisons p-values <0.05 were considered significant.

## Results

### Literature search

**Fig 1:**
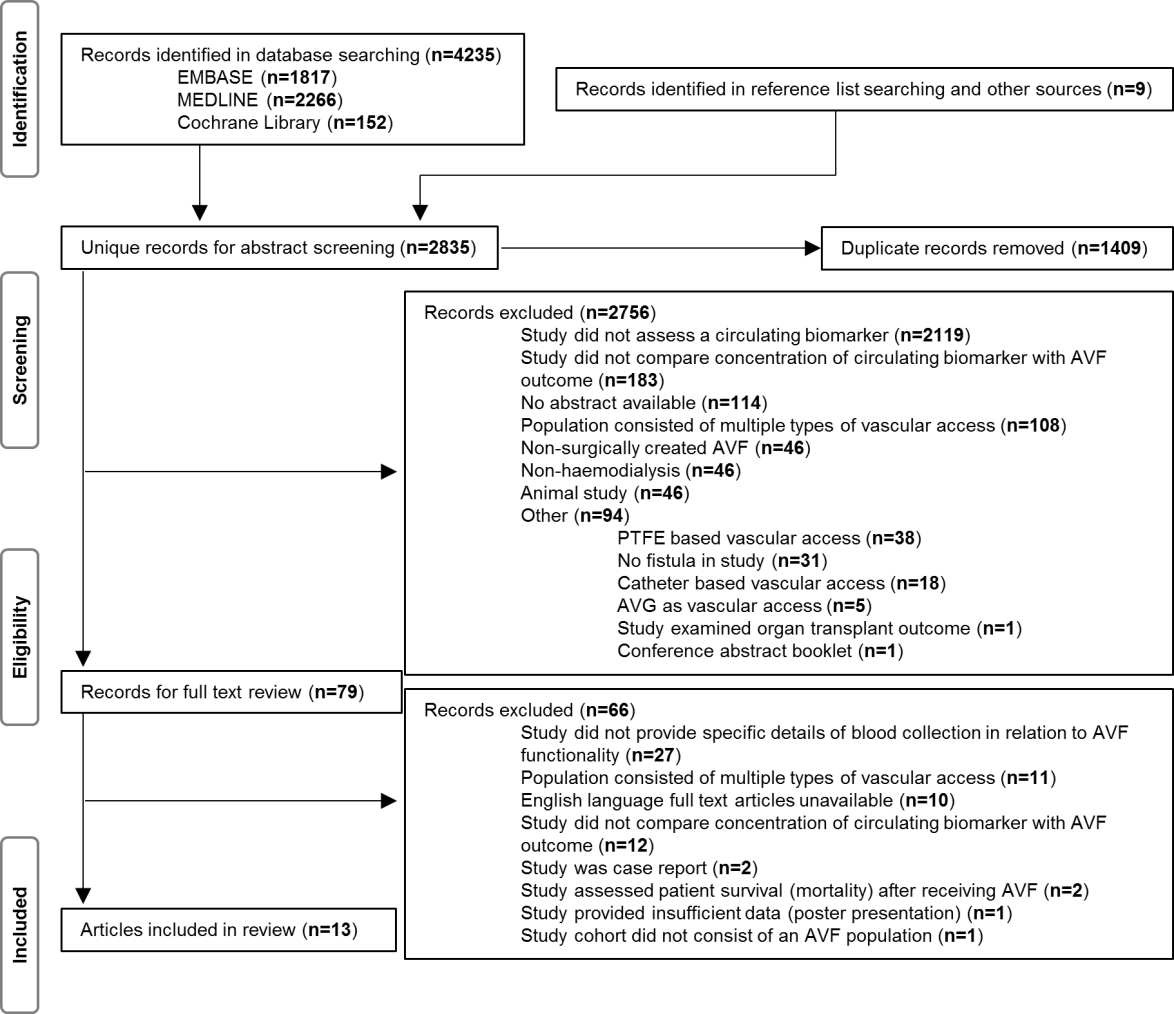
PRISMA study selection flow chart.

Initial database searches yielded 4235 potentially eligible papers for inclusion. A further nine studies were identified from eligible reference lists (Fig 1). After removing 1409 duplicates, 2835 unique abstracts were screened. Of these, 2756 were excluded, mainly because they did not assess a circulating biomarker, and the full text of 79 studies were assessed. Sixty six studies were excluded after reviewing the full text, primarily due to failure to specify the timing of blood collection relative to AVF failure. A total of thirteen studies satisfied the eligibility criteria and were included in this review (Fig 1) [8–20].

### Study characteristics and risk of bias

The characteristics of the included studies are summarised in Table 1. Nine of the studies adopted a longitudinal cohort design (8 prospective [8, 10, 12–14, 17, 19, 20] and 1 retrospective [16]), the remaining four were cross-sectional case-control studies [9, 11, 15, 18]. Of the thirteen studies, only three adjusted their results for the VA risk factors of age and sex [10, 16, 19]. Only five studies collected blood samples for biomarker measurement at a time considered appropriate to the primary outcome assessed, i.e. for studies assessing the future risk of AVF complications, blood collection within one week of AVF creation was deemed appropriate, and for studies assessing biomarkers associated with the presence of an AVF complication, blood collection within one week of the time of AVF failure [8–10, 14, 19]. All but four studies included an AVF population representative of the general HD population (e.g. excluded co-morbidities such as active infection) [8, 12, 18, 19], and only one failed to provide clear patient selection criteria [17].

**Table 1:** Characteristics of the included studies.

Reference	No. Patients	Study Location	Study Design	AVF failure due to	Method(s) of diagnosis of AVF failure	Time of Blood Collection	History of AVF functionality	AVF Location (Case vs Control)
Baumann 2003 [8]	N = 62 Cases:24 Controls:38	Germany	Cohort	Thrombosis within 30 days of AVF creation	Ultrasound and/or Surgery	Before AVF creation	Newly created AVF	n/r
Bilgic 2015 [9]	N = 94 Cases:51 Controls:43	Turkey	Case Control	Stenosis	Ultrasound and Fistulogram	At AVF failure	Functional for at least 6 months prior to analysis (cases and controls)	n/r
Bojakowski 2012 [10]	N = 45 Cases:11 Controls:34	Poland	Cohort	Stenosis and/or thrombosis within 12 weeks of AVF creation	Ultrasound and Angiography	At AVF creation	Newly created AVF. AVF patent for 52 weeks post creation (controls)	RC–(100% vs 100%)
Candan 2014 [11]	N = 80 Cases:42 Controls:38	Turkey	Case Control	Thrombosis	Ultrasound and/or Fistulogram	Collected prior to a mid-week dialysis treatment	Newly created AVF, functional for 3 months prior to failure (cases). Functional AVF for over 3 years post creation (controls)	n/r
Gagliardi 2011 [12]	N = 91 Cases:37 Controls:54	Italy	Cohort	Thrombosis	Access blood flow monitoring	Collected at monthly intervals	Functional with no pre-existing vascular abnormalities (cases and controls)	BC–(100% vs 100%)
Jaberi 2007 [13]	N = 58 Cases:18 Controls:40	Canada	Cohort	Cephalic arch stenosis	Fistulogram	Variable times within 6 months of failure diagnosis	Unclear	BC–(94% vs 70%)
Kaygin 2013 [14]	N = 386 Cases:75 Controls:311	Turkey	Cohort	Failure to mature	Dialysis complications	At AVF creation	AVF failure within first 12 weeks (cases). AVF patent at end of 12 weeks (control)	RC–(59% vs 69%) BC–(41% vs 30%)
Kim 2013 [15]	N = 64 Cases:34 Controls:30	Korea	Case Control	Stenosis	Ultrasound	Prior to midweek dialysis at monthly intervals for a total of 6 months	Functional with no pre-existing abnormalities for at least 6 months (cases and controls)	RC–(100% vs 100%)
Kirkpantur 2008 [16]	N = 99 Cases:38 Controls:61	Turkey	Cohort	Thrombosis	Dialysis complicationsand Angiography	At AVF creation plus fasting monthly pre-HD collections up to failure (cases) or end of follow up (controls)	AVF were patent for at least 6 weeks following surgical opening of AVF (cases and controls)	RC–(76% vs 77%) BC–(24% vs 21%)
Masaki 1999 [17]	N = 184 Cases:83 Controls:101	Japan	Cohort	Stenosis or Thrombosis	Surgery and/or Radiography and/or Ultrasound	At AVF failure (cases), unspecified for controls	n/r	n/r
Ozdemir 2005 [18]	N = 141 Cases:60 Controls:81	Turkey	Case Control	Thrombosis	n/r	6 months prior to AVF thrombosis (cases) or 6 months prior to study (controls)	One or more thromboses (cases). No recorded thromboses (controls)	SB–(30.8% vs 4.9%)
Wu 2009 [19]	N = 100 Cases:41 Controls:59	China	Cohort	Restenosis following PTA	Fistulography	Immediately before PTA (cases) or routine HD (controls)	All patients had history of stenosis (cases and controls) and underwent PTA	n/r
Yilmaz 2014 [20]	N = 108 Cases:64 Controls:44	Turkey	Cohort	Stenosis	Ultrasound and Angiography	Measured 6 months prior to stenosis diagnosis	Functional for at least 6 months prior to analysis (cases and controls)	n/r

AVF: arteriovenous fistula; n/r: not recorded; HD: haemodialysis; RC: radiocephalic AVF; BC: brachiocephalic AVF. SB: Snuffbox AVF; PTA: percutaneous transluminal angioplasty. Studies by Baumann *et al*. and Masaki *et al*. were not included in the meta-analysis as neither study presented the mean concentration of measured biomarker(s) within the text.

Using the modified Ottawa-Newcastle tool to assess the risk of bias in the nine cohort studies (S3 File) we found one with a very low risk [10], 3 studies with a low risk [8, 12, 14], 2 studies with a medium risk [16, 17] and the final 3 with a high risk of bias [13, 19, 20]. Of the case control studies, 2 were considered low risk [9, 11] and 2 were considered medium risk [15, 18] of bias (S4 File).

The combined population of the thirteen studies totalled 1512 participants, including 578 cases and 934 controls. Total sample size ranged from 45 to 386 patients [10, 14]. The median number of cases per study was 40 (range 11 to 83), with a median age of 57 years (range 45 to 68) and comprising a median 55.8% males (range 36.6 to 65.2). The median number of controls per study was 44 (range 30 to 311), with a median age of 55 years (range 41 to 62) and comprising a median 56.7% males (range 45.8 to 70.6).

All studies, excluding that of Baumann *et al*, and Masaki *et al*., listed mean biomarker concentrations for groups of patients with failed (cases) or patent (controls) AVFs [8, 17]. The study by Bojakowski *et al*. compared groups of patients who had early fistula failure (12 weeks post AVF creation), late fistula failure (52 weeks post AVF creation) or no fistula failure [10]. For the purposes of this review, patients with early fistula failure were considered to be the cases as failure occurred closer to the time of blood collection (at time of AVF creation), and patients with late AVF failure were excluded [10]. Five studies included only patients with a newly formed fistula in anticipation of HD [8, 10, 11, 14, 16], while the remaining studies included only patients receiving routine HD [9, 12, 13, 15, 17–20]. In all but one study, control patients included in this review had an AVF that was patent for at least 6 months. In contrast, all patients included in the study by Wu *et al*., had received a percutaneous transluminal angioplasty (PTA) to resolve a previous AVF dysfunction and controls were considered those patients who did not experience restenosis following this procedure [19].

### Reported biomarkers and AVF failure

We observed considerable inter-study variation in the definition of AVF failure. For the purpose of this meta-analysis we initially included all outcomes reported (stenosis, thrombosis and AVF dysfunction arising from unknown complications which led to HD complications) as AVF failure (Table 1). Six studies investigated the association of circulating biomarkers with AVF stenosis or restenosis [9, 13, 15, 16, 19, 20] four studies with AVF thrombosis [8, 11, 12, 18], two with a mixture of both AVF stenosis and thrombosis [10, 17], and one study did not specify the cause of AVF dysfunction [14]. There was considerable disparity in the timing of blood sample collection relative to AVF failure between studies (Table 1). Three studies provided mean biomarker values over time, often including samples taken at the time of AVF creation, as well as samples leading up to and including AVF dysfunction [12, 15, 16]. Three studies analysed blood samples before or at the time of fistula creation only [8, 10, 15], and four at the time of AVF failure only [9, 11, 17, 19]. The final three studies collected blood samples within the 6 months prior to AVF failure [13, 18, 20]. Most studies lacked specific details of the methods of biomarker measurement, with many stating quantification was achieved by an “automated analyser” (S1 Table). Furthermore it was not well reported whether biomarkers were measured in plasma, serum or whole blood (S1 Table). For the purpose of the meta-analysis, biomarker data from only 11 of the thirteen studies were included as Baumann *et al* and Masaki *et al* did not provide mean biomarker concentrations for patients that did and did not have AVF failure [8, 17].

### Comparison of biomarker concentrations in cases and controls

All biomarkers assessed in the reviewed studies are shown in S1 Table. Out of the 48 measured biomarkers, 12 (albumin, creatinine, C-reactive protein (CRP), calcium, ferritin, haemoglobin, high-density lipoprotein (HDL-C) cholesterol, low-density lipoprotein (LDL-C) cholesterol, parathyroid hormone, phosphorus, total cholesterol (TC) and triglycerides (Table 2)) satisfied the inclusion criteria for the current meta-analysis. The number of studies assessing each biomarker varied from 10 studies (albumin) to three studies (creatinine). Mean concentrations of the biomarkers in case and control groups reported by each study are listed in Table 3. There was considerable intra- and inter-study variation in the number of decimal places reported for each biomarker concentration and thus all biomarkers are listed to the decimal point as originally published. Further, for all but one paper (Jaberi *et al*., [13]), biomarker concentrations were reported in United States standard (US) units and so to avoid decimal point rounding errors, the subsequent meta-analyses were performed using the concentrations reported in US units. Data from Jaberi *et al*., was converted from International Standard (SI) units into US units for inclusion into the meta-analysis [13]. For the purpose of clarity, biomarker concentrations are presented in both US and SI units in Table 3. It is important to note that due to the often interchangeable use of the term phosphorus and phosphate between studies, data relating to either terminology were included in the meta-analysis denoted here as ‘phosphorus’ [21].

**Table 2:** Biomarkers for which meta-analyses were performed.

Biomarker	No. of studies	References	Total population
Albumin	10	9–16, 19, 20	1125
Calcium	5	9, 13, 15, 19, 20	424
Creatinine	3	10, 14, 19	531
CRP	9	9–12, 14, 15, 18–20	1109
Ferritin	5	9–11, 18, 20	468
Haemoglobin	6	9–11, 13, 16, 20	484
HDL-C	7	9–11, 14, 16, 19, 20	912
LDL-C	7	9–11, 14, 16, 19, 20	912
PTH	4	9, 11, 18, 20	423
Phosphorus	5	9, 13, 15, 19, 20	424
Total cholesterol	4	11, 12, 14, 16	656
Triglycerides	7	9–11, 14, 16, 19, 20	912

CRP: C-reactive peptide; HDL-C: High density lipoprotein cholesterol; LDL-C: Low density lipoprotein cholesterol; PTH: Parathyroid hormone.

**Table 3:** Biomarker concentrations in patients with (case) and without (control) AVF failure

Marker US units (SI units)	ref	Cases			Controls			*p*
		n	Mean	SD	n	Mean	SD	
Albumin g/dL (g/L)	9	51	3.87 (38.7)	0.36 (3.6)	43	3.91 (39.1)	0.42 (4.2)	0.189
	10	11	3.2 (32)	1.1 (11)	34	4.0 (40)	0.4 (4)	dns
	11	42	3.8 (38)	0.3 (3)	38	3.8 (38)	0.3 (3)	0.924
	12	37	3.26 (32.6)	0.40 (4.0)	54	3.49 (34.9)	0.46 (4.6)	**0.018**
	13	18	3.4 (34)	0.4 (4)	40	3.3 (33)	0.4 (4)	n/r
	14	75	3.0 (30)	0.8 (8)	311	3.96 (39.6)	0.4 (4)	**<0.001**
	15	34	3.8 (38)	3.5 (35)	30	3.9 (39)	2.2 (22)	0.761
	16	38	3.67 (36.7)	0.26 (2.6)	61	3.95 (39.5)	0.39 (3.9)	**0.010**
	19^1^	41	3.59 (35.9)	0.44 (4.4)	59	3.63 (36.3)	0.43 (4.3)	0.64
	20	64	3.76 (37.6)	0.68 (6.8)	44	3.73 (37.3)	0.56 (5.6)	**0.005**
Calcium mg/dL (mmol/L)	9	51	8.40 (2.10)	0.52 (0.13)	43	8.50 (2.13)	0.65 (0.16)	0.231
	13	18	9.16 (2.29)	0.60 (0.15)	40	9.08 (2.27)	0.72 (0.18)	n/r
	15	34	8.5 (2.1)	4.1 (1.0)	30	8.6 (2.2)	2.7 (0.7)	0.719
	19	41	9.97 (2.49)	1.04 (0.26)	59	9.89 (2.47)	0.86 (0.21)	0.66
	20	64	8.02 (2.00)	0.64 (0.16)	44	8.10 (2.02)	0.55 (0.14)	0.422
Creatinine mg/dL (μmol/L)	10	11	5.2 (459.7)	1.9 (168.0)	34	5.1 (450.8)	1.9 (168.0)	dns
	14	75	4.2 (371.3)	2.6 (229.8)	311	4.1 (362.4)	2.5 (221.0)	0.597
	19	41	10.60 (937.04)	2.20 (194.48)	59	10.20 (901.68)	2.20 (194.48)	0.34
CRP mg/L (nmol/L)	9	51	18.77 (178.77)	20.48 (195.05)	43	13.28 (126.48)	12.47 (118.76)	0.271
	10	11	18.6 (177.15)	16.8 (160.0)	34	7.3 (69.5)	6.6 (62.9)	dns
	11	42	12.6 (120.0)	16.6 (158.1)	38	12.4 (118.1)	16.3 (155.24)	0.940
	12^2^	37	11.98 (114.10)	9.1 (86.7)	54	9.83 (93.62)	11.4 (108.6)	0.341
	14	75	18.6 (177.2)	4.3 (41.0)	311	4.6 (43.8)	2.2 (21.0)	**<0.001**
	15	34	3.8 (36.2)	13.4 (127.6)	30	4.0 (38.1)	15.3 (145.72)	0.479
	18	60	12.9 (122.9)	15.0 (142.9)	81	11.2 (106.7)	11.4 (108.6)	n/r
	19	41	7.3 (69.5)	9.1 (86.7)	59	8.8 (83.8)	10.0 (95.2)	0.44
	20	64	9.75 (92.86)	11.97 (114.00)	44	8.94 (85.1)	12.3 (117.2)	0.502
Ferritin ng/mL (pmol/L)	9	51	422.4 (949.1)	240.8 (541.1)	43	439.1 (986.7)	230.7 (518.4)	0.346
	10	11	170.4 (382.9)	104.7 (235.3)	34	235.7 (529.6)	314.5 (706.7)	dns
	11	42	855.1 (1921.4)	714.9 (1606.4)	38	890.6 (2001.2)	619.1 (1391.1)	0.814
	18	60	552.4 (1241.2)	821.6 (1846.1)	81	497.6 (1118.1)	308.4 (693.0)	n/r
	20	64	542.43 (1218.77)	230.45 (517.8)	44	539.15 (1211.47)	286.37 (643.47)	0.657
Hb g/dL (g/L)	9	51	10.85 (108.5)	1.15 (11.5)	43	10.90 (109.0)	1.26 (12.6)	0.542
	10	11	9.7 (97)	1.0 (10)	34	10.9 (109)	1.5 (15)	dns
	11	42	11.6 (116)	1.5 (15)	38	11.3 (113)	1.3 (13)	0.266
	13	18	11.6 (116)	1.5 (15)	40	11.6 (116)	1.3 (13)	n/r
	16	38	10.9 (109)	1.0 (10)	61	11.2 (112)	1.0 (10)	0.080
	20	64	10.83 (108.3)	1.97 (19.7)	44	10.75 (107.5)	1.82 (18.2)	0.848
HDL-C mg/dL (mmol/L)	9	51	39.6 (1.0)	10.3 (0.3)	43	38.7 (1.0)	11.2 (0.3)	0.305
	10	11	52.1 (1.4)	18.5 (0.5)	34	56.7 (1.5)	17.6 (0.5)	dns
	11	42	33.9 (0.9)	13 (0.3)	38	32.4 (0.8)	8.9 (0.2)	0.552
	14	75	42.8 (1.1)	12.5 (0.32)	311	39.6 (1.0)	11.8 (0.3)	n/r
	16	38	31.4 (0.8)	4.4 (0.1)	61	44 (1.1)	7 (0.2)	**0.015**
	19	41	54 (1.4)	19 (0.5)	59	50 (1.3)	17 (0.4)	0.24
	20	64	31.8 (0.8)	12.6 (0.3)	44	51.5 (1.3)	11.9 (0.3)	**<0.001**
LDL-C mg/dL (mmol/L)	9	51	154.5 (4.0)	32.6 (0.8)	43	128.7 (3.3)	28.6 (0.74)	**<0.001**
	10	11	108.6 (2.8)	48.1 (1.3)	34	99.5 (2.6)	45.7 (1.2)	dns
	11	42	98 (2.5)	35.1 (0.9)	38	95.9 (2.5)	33 (0.9)	0.784
	14	75	118.7 (3.1)	28.6 (0.7)	311	114.8 (3.0)	28.3 (0.7)	n/r
	16	38	62.8 (1.6)	11.0 (0.3)	61	97.4 (2.5)	19 (0.5)	**0.022**
	19	41	113 (2.9)	33 (0.9)	59	102 (2.6)	30 (0.8)	0.09
	20	64	102.69 (2.66)	36.13 (0.94)	44	99.86 (2.59)	39.49 (1.02)	0.378
PTH pg/mL (= ng/L)	9	51	332.8	160.5	43	319.5	204.3	0.105
	11^3^	42	267.5	229.5	38	311.4	316.1	0.477
	18	60	449.4	363.4	81	492.0	409.9	n/r
	20	64	371.70	301.04	44	361.57	327.48	0.815
Phosphorus mg/dL (mmol/L)	9	51	5.80 (1.87)	1.90 (0.61)	43	5.75 (1.86)	1.79 (0.58)	0.365
	13^4^	18	6.25 (2.02)	2.20 (0.71)	40	5.14 (1.66)	1.46 (0.47)	n/r
	15	34	5.3 (1.7)	26.2 (8.46)	30	5.2 (1.7)	26.8 (8.7)	0.813
	19^4^	41	4.39 (1.42)	1.36 (0.44)	59	4.45 (1.44)	1.74 (0.56)	0.84
	20	64	6.29 (2.03)	1.51 (0.49)	44	6.13 (1.98)	1.49 (0.48)	0.375
TC mg/dL (mmol/L)	11	42	167 (4.3)	45.3 (1.2)	38	160.7 (4.2)	43.7 (1.1)	0.544
	12	37	148.24 (3.84)	23.50 (0.61)	54	136.00 (3.52)	35.60 (0.92)	0.069
	14	75	183.5 (4.8)	28.2 (0.7)	311	172.3 (4.5)	44.9 (1.2)	n/r
	16	38	145.6 (3.8)	35.1 (0.9)	61	155 (4)	34.5 (0.9)	0.860
TG mg/dL (mmol/L)	9	51	279.5 (3.2)	90.4 (1.0)	43	288.9 (3.3)	106.6 (1.2)	0.411
	10	11	158.4 (1.8)	75.8 (0.9)	34	148.6 (1.7)	82.4 (0.9)	dns
	11	42	181.8 (2.1)	84.7 (1.0)	38	170.1 (1.9)	84.7 (1.0)	0.891
	14	75	148.6 (1.7)	74.8 (0.9)	311	153.8 (1.7)	82.2 (0.9)	n/r
	16	38	143.6 (1.6)	66.2 (0.8)	61	161.3 (1.8)	61.5 (0.7)	0.390
	19	41	175 (2.0)	105 (1.2)	59	155 (1.8)	80 (0.9)	0.27
	20	64	195.58 (2.2)	89.41 (1.0)	44	198.78 (2.3)	96.436 (1.09)	0.865

CRP: C-reactive peptide; Hb: Haemoglobin; HDL-C: High density lipoprotein cholesterol; LDL-C: Low density lipoprotein cholesterol; PTH: Parathyroid hormone; TC: Total cholesterol; TG: Triglycerides. There was considerable inter and intra-study variability in the number of decimal places reported for each biomarker observed and to avoid ambiguity, data are shown exactly as reported by the original studies. Biomarker concentrations are reported in both US and SI units (the latter in parentheses). P-values reported as published, those in bold are considered significant. Those p-values not reported denoted as n/r, those p-values not reported due to ANOVA analyses denoted as dns (did not specify). n = patient number; SD: standard deviation; AVF: arteriovenous fistula. Gagliardi *et al* [12] and Kaygin *et al* [14] did not specify SD or standard error of the mean (SEM), however based on reported p-values it was assumed to be SD. Kim *et al* [15] reported SEM, therefore SD was manually calculated using the formula: $SD=SEM\times \sqrt{n}.$ ^1^Values reported by [19] as mg/dL, however this would make the albumin values 1000-fold different from the other reported values, therefore assumed to be g/dL. ^2^Values reported by [12] as ng/mL, however this would make the CRP values 1000-fold different from the other reported values, therefore assumed to be mg/L. ^3^Values reported by [11] as pg/dL, however this would make the PTH values 100-fold different from the other reported values, therefore assumed to be pg/mL. ^4^Values reported by [13] and [19] are reported as phosphate measurements, however the terms phosphorus and phosphate are often interchangeably used in clinical reports [21] and therefore all measurements were considered to be phosphorus.

### Meta-analysis findings

#### The association of biomarkers with all AVF failure

The association of the eligible 12 biomarkers with AVF failure was assessed using an inverse-variance random-effects model. None of the twelve biomarkers were found to be significantly associated with AVF failure when we included all measured outcomes of AVF failure (Table 4, S1 Fig). A leave-one-out sensitivity analysis was performed for each of the twelve biomarkers (S5 File). Only the exclusion of the data by Kirkpantur *et al*. led to statistically significant inverse associations of elevated LDL-C and TC with AVF failure (p = 0.02 and p = 0.007 respectively) [16]. High I^2^ values were observed for most meta-analyses when including all eligible studies (Table 4), with little change observed during most of the leave-one-out analyses (S5 File).

**Table 4:** Meta-analysis of biomarker data in relation to AVF failure.

Biomarker	Studies	N_Cases_	N_Controls_	SMD (95%CI)	*p* value*	I^2^	I^2^ *p* value
Albumin	10	411	714	-0.44 [-0.95, 0.07]	0.09	93%	**< 0.001**
Calcium	5	208	216	-0.04 [-0.24, 0.15]	0.67	0%	0.86
Creatinine	3	127	404	0.08 [-0.13, 0.28]	0.46	0%	0.84
CRP	9	415	694	0.75 [-0.32, 1.82]	0.17	98%	**< 0.001**
Ferritin	5	228	240	-0.01 [-0.19, 0.18]	0.92	0%	0.92
Haemoglobin	6	224	260	-0.10 [-0.33, 0.14]	0.42	36%	0.17
HDL-C	7	322	590	-0.45 [-1.12, 0.23]	0.20	95%	**< 0.001**
LDL-C	7	322	590	-0.06 [-0.64, 0.53]	0.85	93%	**< 0.001**
PTH	4	217	206	-0.04 [-0.23, 0.15]	0.67	0%	0.83
Phosphorus	5	208	216	0.10 [-0.10, 0.30]	0.32	4%	0.39
TC	4	192	464	0.14 [-0.12, 0.41]	0.28	50%	0.11
TG	7	322	590	-0.02 [-0.17, 0.12]	0.74	0%	0.70

SMD: standardised mean difference; I^2^: heterogeneity index; N_Cases_: Number of patients with a failed AVF; N_Controls_: Number of patients with a patent AVF; CRP: C-reactive protein; HDL-C: High density lipoprotein cholesterol; LDL-C: Low density lipoprotein cholesterol; PTH: Parathyroid hormone; TC: Total cholesterol; TG: Triglycerides. P-values in bold are significant.

*Calculated according to inverse-variance random-effects model

#### The association of biomarkers with AVF thrombosis or stenosis

**Fig 2:**
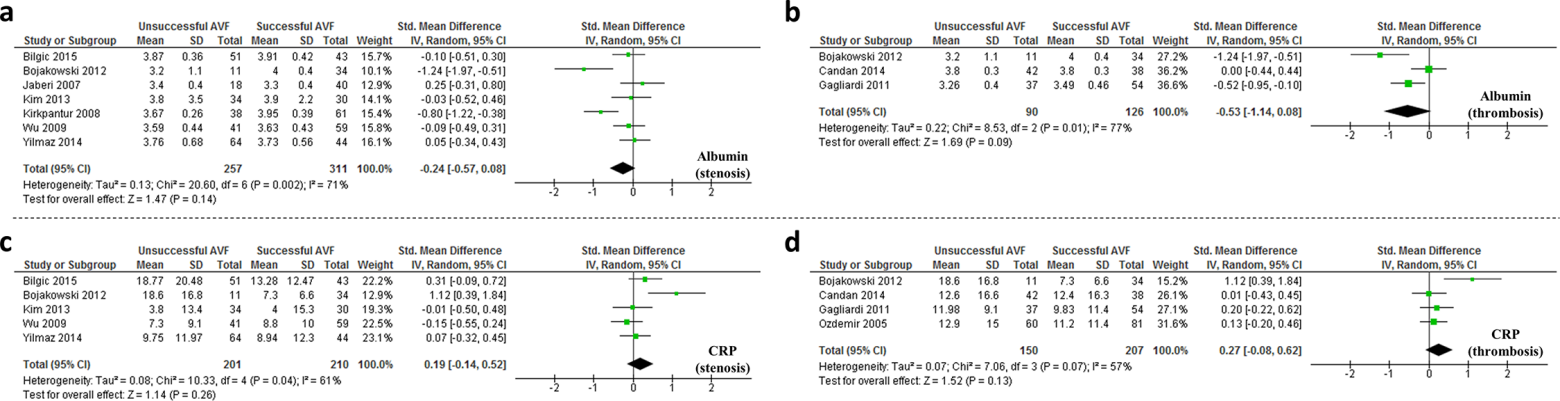
Circulating levels of albumin or CRP are not significantly associated with either AVF stenosis or AVF thrombosis in HD patients. Forest plot of meta-analysis data showing the association between circulating albumin with AVF (a) stenosis or (b) thrombosis; and circulating CRP with AVF (c) stenosis or (d) thrombosis.

Sub-analyses were conducted to specifically assess the association of biomarkers with AVF stenosis/restenosis [9, 13, 15, 16, 19, 20] or AVF thrombosis (Table 1) [11, 12, 18]. The study by Kaygin *et al*, was not clear in its definition of AVF failure [14], and was therefore excluded from these analyses. In line with the inclusion criteria for meta-analysis (assessed in at least 3 studies and ≥100 participants), albumin and CRP were the only 2 biomarkers which could be assessed in these sub-analyses (Fig 2). No significant association of either of these markers with AVF stenosis or thrombosis was observed. It was noted that the cases reported by Bojakowski *et al*. included a mixture of patients with failed AV due to either thrombosis or stenosis [10]. To eliminate potential confounding from this mixed population, meta-analyses assessing the association of albumin and CRP with AVF stenosis, and CRP with AVF thrombosis were repeated, excluding data from Bojakowski *et al*. [10]. No association of these markers with either AVF outcome was observed (Table 5). We were unable to assess the association of albumin with AVF thrombosis as only 2 studies remained after excluding data from Bojakowski and colleagues which violated inclusion criteria for the current study. A leave-one-out sensitivity analysis was also performed for each biomarker (S5 File).

**Table 5:** Meta-analysis of the association of albumin and CRP with AVF stenosis and thrombosis.

AVF Outcome	Biomarker	Studies	N_Cases_	N_Controls_	SMD (95%CI)	*p* value*	I^2^	I^2^ *p-*value
Stenosis	Albumin	7	257	311	-0.24 [-0.57, 0.08]	0.14	71%	**0.002**
	Albumin†	6	246	277	-0.14 [-0.42, 0.15]	0.34	60%	**0.03**
	CRP	5	201	210	0.19 [-0.14, 0.52]	0.26	61%	**0.04**
	CRP†	4	190	176	0.06 [-0.15, 0.26]	0.59	0%	0.44
Thrombosis	Albumin	3	90	126	-0.53 [-1.14, 0.08]	0.09	77%	**0.01**
	CRP	4	150	207	0.27 [-0.08, 0.62]	0.13	57%	0.07
	CRP†	3	139	173	0.12 [-0.10, 0.34]	0.30	0%	0.83

SMD: standardised mean difference; I^2^: heterogeneity index; CRP: C-reactive protein; N_Cases_: Number of patients with a failed AVF; N_Controls_: Number of patients with a patent AVF

*Calculated according to inverse-variance random-effects model

†Analysis excluded mixed population data from Bojakowski *et al*. P-values in bold are significant.

## Discussion

### Main Findings

AVF failure is a significant cause of morbidity and expense in the HD population [2]. Older age, female sex, diabetes, and smaller vein calibre are established risk factors for AVF failure [22]. No comprehensive assessment of the association between circulating biochemical factors and AVF failure has been previously published. We analysed data from thirteen studies and included twelve biomarkers in a meta-analysis from a possible group of 48 [8–20]. There was no significant association between any of these 12 biomarkers with AVF failure due to any cause, or when AVF failure was specifically due to stenosis or thrombosis. A possible reason for this result is study heterogeneity, evidenced by high I^2^ statistic and significant I^2^ p-value in many analyses.

### Sources of Heterogeneity

The studies included in this review varied in many aspects. One such variation is differences between the methods used to measure biomarker concentrations. Most studies failed to provide comprehensive details on laboratory methods and the medium in which biomarkers were quantified (e.g. plasma or serum), and for those that did provide this information, blood medium varied considerably between studies. Differences in methodology have also previously been acknowledged to generate heterogeneity of biomarker concentrations in other studies [19, 23]. Another source of heterogeneity is the timing in which blood samples were collected in relation to AVF assessment. Of the eleven studies included in the meta-analyses, only three clearly stated that blood collection occurred at the time of AVF failure [9, 11, 19]. The remainder took a single measurement at AVF creation [10, 14], a single measurement while the AVF was functional [18, 20], or collected several blood samples to generate a mean over time [12, 13, 15, 16]. These methods could potentially either precede biochemical changes associated with AVF failure or combine circulating biochemical parameters associated with patent AVFs with those of failing AVFs, thereby dampening any possible association. Thus few of the studies were appropriately designed to identify circulating markers of AVF failure.

The primary outcome assessed also varied amongst the included studies. For example, Gagliardi *et al*. investigated factors that influenced AVF failure due to thrombosis, whereas Jaberi *et al* investigated the factors influencing cephalic arch stenosis [12, 13]. Whilst both studies presented results in a way that allowed an association to be drawn between a biomarker and AVF outcome, in reality the two populations themselves represent significantly different cohorts.

In an attempt to overcome limitations of inter-study heterogeneity, sub analyses were conducted to assess the association of biomarkers with AVF stenosis or AVF thrombosis specifically. Due to our meta-analysis inclusion criteria this analysis was limited to albumin and CRP. There was no statistically significant association found between either albumin or CRP with AVF stenosis or thrombosis. These sub-analyses are likely underpowered although heterogeneity appeared to be reduced since the I^2^ statistics were lower than for analyses of all studies.

The location of the AVF differed between studies, although most investigations focussed on brachiocephalic or radiocephalic fistulae. Brachiocephalic fistulae are reported to have greater patency, although they are also associated with a greater incidence of complications, particularly steal syndrome [24–26]. Most studies provided little information on the follow-up time, length of time patients were on HD prior to the study, history of previous AVF events, prevalence of diabetes and medication usage, which are important determinants of AVF outcome [27]. Overall the identified studies failed to report important and well defined determinants of AVF outcome. The quality of clinical research performed in this area may be greatly improved by standardised definitions of parameters that should be included in such studies in order to guide future work.

### Future Directions

Six potentially important biomarkers were not included in this study as they did not fulfil the specified inclusion criteria. Elevated fibrinogen has been reported to be significantly associated with AVF failure [14], although this is contradicted by another study where no association was found between fibrinogen and AVF failure [12]. Red blood cell distribution width (RDW), an indicator of anisocytosis, was reported to be significantly greater in patients with AVF failure in a single study [10]. Elevated RDW is also associated with other cardiovascular conditions such as coronary artery disease and myocardial infarction [28, 29], suggesting that increased RDW alone is unlikely to be specific to AVF failure. Plasma asymmetrical dimethylarginine (ADMA), an endogenous inhibitor of nitric oxide synthase, has also reported to be higher in patients with restenosis following a PTA, than patients whose AVF remained patent following the procedure [19]. Similarly, elevated levels of serum osteoprotegerin (OPG) have been reported to predict AVF stenosis [15], possibly linked to the histopathological similarities between AVF stenosis and atherosclerosis [12]. Soluble endothelial leukocyte adhesion molecule-1 (sE-selectin) has also been reported to be significantly elevated in patients with AVF stenosis [9]. E-selectin has been previously implicated in intimal hyperplasia [9]. Finally, significantly lower levels of the angiogenic cytokine vascular endothelial growth factor A (VEGF-A) were reported in patients with AVF thrombosis [11]. VEGF-A has been shown in animal models to have an anti-thrombotic effect and therefore may be a valuable prognostic tool [11]. Additional studies will be needed to conclusively establish the relationship between AVF functionality and these six biomarkers, however these data provide new directions for pathophysiological investigations into the failing AVF.

### Study Limitations

This review had a number of limitations. Firstly, few data have been published in this field and thus sample sizes in our meta-analyses were small, reducing our analytical power. We set our eligibility for meta-analysis at biomarkers assessed in ≥3 studies with a total population of ≥100 participants, which we felt was a minimum requirement for such an analysis. As such we have excluded some biomarkers from the meta-analysis. Consequently, this also limited our ability to perform sub-analyses with regards to a specific cause of AVF failure (e.g. AVF stenosis or thrombosis) for all biomarkers assessed in this meta-analysis. It is plausible that analysing a composite outcome (all AVF failure) may mask the effect of biomarkers on a specific cause of AVF failure. Secondly, there was significant heterogeneity between the studies, impacting on the strength of findings in the meta-analyses. Thirdly, AVF is just one form of VA and as such we have excluded a large proportion of studies that investigated the outcome of arteriovenous grafts and other forms of VA. However, an AVF is generally recognised as the superior form of VA and this was a motivating factor in our study design. Fourthly, we obtained data from publically available literature and therefore did not have access to primary data. In instances where data were unavailable, authors were contacted to obtain relevant data. We cannot exclude the potential influence of publication bias on our findings. Finally, we were limited to searching for articles published in the English language, and it is therefore possible that potentially useful papers detailing other markers of AVF failure in non-English journals were not included here. We acknowledge this as a potential source of bias in the findings of our analysis.

## Conclusion

To our knowledge this meta-analysis represents the first comprehensive investigation of biomarkers associated with AVF failure. Our results demonstrate no conclusive association of any previously assessed biomarker with AVF failure, although it is important to note that the range of evaluated biomarkers is narrow and predominantly restricted to markers assessed in routine clinical investigations. We conclude that rigorously designed studies of biologically plausible biomarkers are needed to decide the clinical value of biomarkers for monitoring HD. Care must be taken during experimental design, to ensure study protocol effectively addresses the primary research question. For example, in investigations designed to correlate biomarkers with AVF failure, blood samples must be taken appropriate to the time of failure.

## Supporting Information

S1 Fig None of the 12 biomarkers were found to be significantly associated with AVF failure. Forest plot of meta-analysis data showing the lack of an association between AVF failure and circulating (a) albumin; (b) calcium; (c) creatinine; (d) C-reactive protein (CRP); (e) ferritin; (f) haemoglobin; (g) high density lipoprotein cholesterol (HDL-C); (h) low density lipoprotein cholesterol (LDL-C); (i) parathyroid hormone (PTH); (j) phosphorus; (k) total cholesterol (TC); and triglycerides.
(PDF)

S2 Fig Circulating levels of albumin or CRP were not significantly associated with AVF stenosis or AVF thrombosis in HD patients, even when data from a mixed population (Bojakowski *et al*.) were removed. Forest plot of meta-analysis data showing the lack of an association between circulating albumin with AVF (a) stenosis; and circulating CRP with AVF (b) stenosis or (c) thrombosis, when data from Bojakowski *et al*. is removed.
(PDF)

S1 File Search strategies. (PDF)

S2 File Data extraction form. (PDF)

S3 File Results of the modified Ottawa-Newcastle tool to assess the risk of bias in cohort studies. (PDF)

S4 File Results of the modified Ottawa-Newcastle tool to assess the risk of bias in case control studies. (PDF)

S5 File Leave-one-out sensitivity tests for all meta-analyses (PDF)

S1 Table Blood collection and laboratory methods used to quantify biomarkers. PTH: parathyroid hormone; HDL-C: high density lipoprotein cholesterol; LDL-C: low density lipoprotein cholesterol; CRP: C-reactive protein; sE-Selectin: soluble E-selectin; eEPCR: soluble endothelial protein C receptor; TG: triglycerides; WBC: white blood cell count; MCV: mean corpuscular volume; MCH: mean corpuscular haemoglobin; RDW: red blood cell distribution width; RBC: red blood cell count; Ca x P: calcium times phosphate; VEGF-A: vascular endothelial growth factor A; MIS: malnutrition inflammation score; CMV: cytomegalovirus; OPG: osteoprotegerin; TC: total cholesterol; ADMA: asymmetrical dimethylarginine; NLR: neutrophil-lymphocyte ratio.
(DOCX)
