# Supplementary figures and images for: A Systematic Review and Meta-Analysis of Circulating Biomarkers Associated with Failure of Arteriovenous Fistulae for Haemodialysis
Source: PLoS One. 2016 Jul 26;11(7):e0159963. doi: 10.1371/journal.pone.0159963 (PMC4961283; doi:10.1371/journal.pone.0159963)

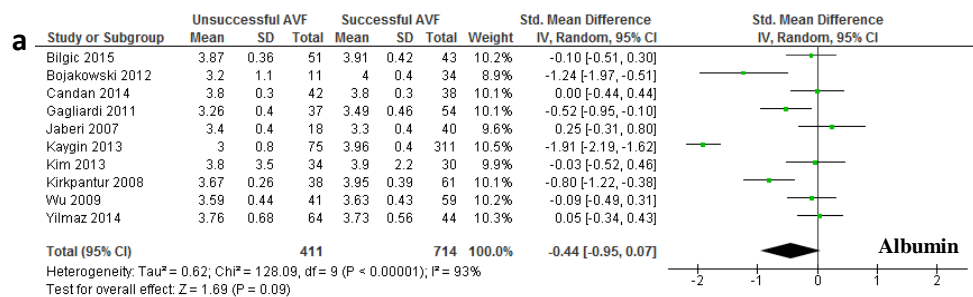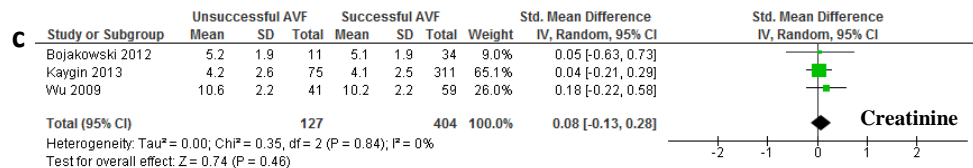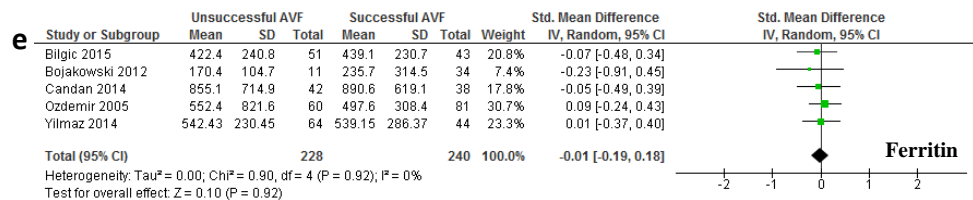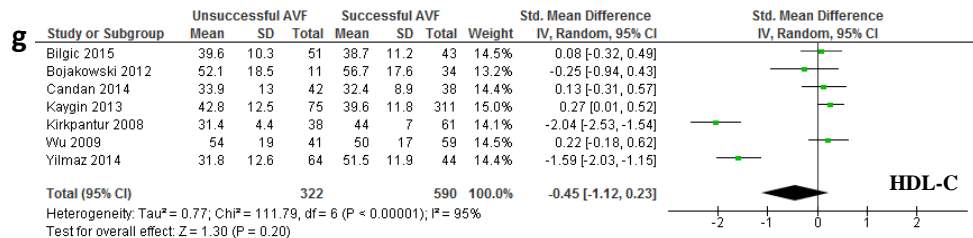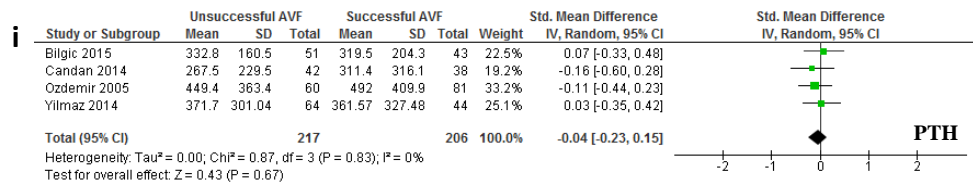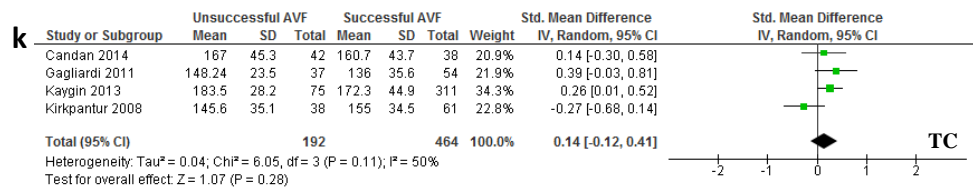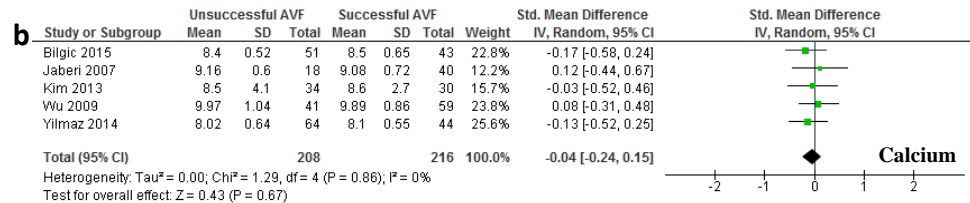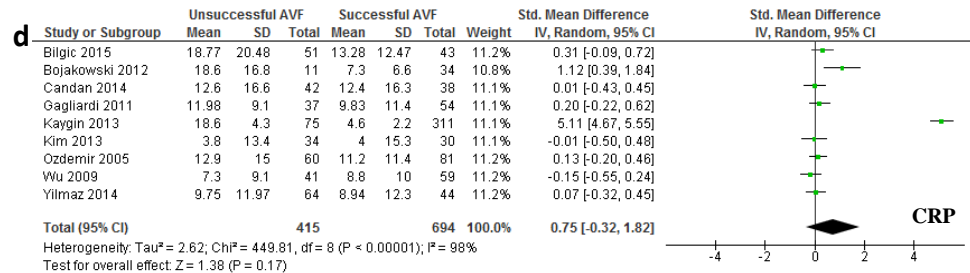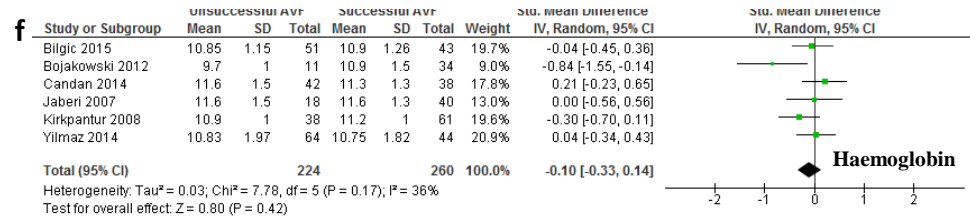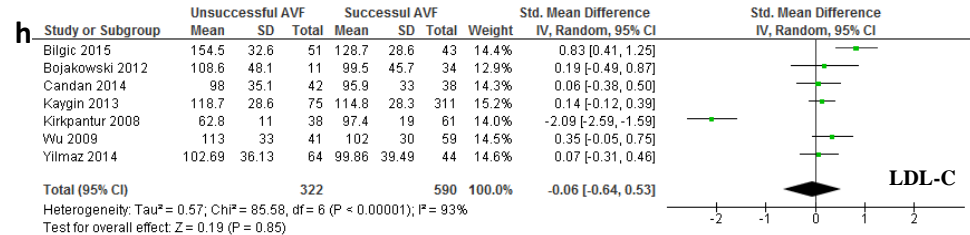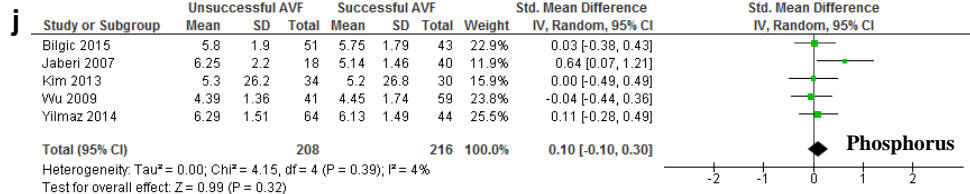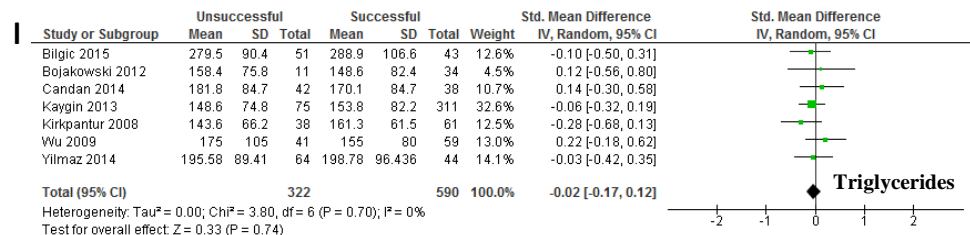

Supplement: S1 Fig — Forest plot of meta-analysis data showing the lack of an association between AVF failure and circulating (a) albumin; (b) calcium; (c) creatinine; (d) C-reactive protein (CRP); (e) ferritin; (f) haemoglobin; (g) high density lipoprotein cholesterol (HDL-C); (h) low density lipoprotein cholesterol (LDL-C); (i) parathyroid hormone (PTH); (j) phosphorus; (k) total cholesterol (TC); and triglycerides. (PDF) [file pone.0159963.s001.pdf]

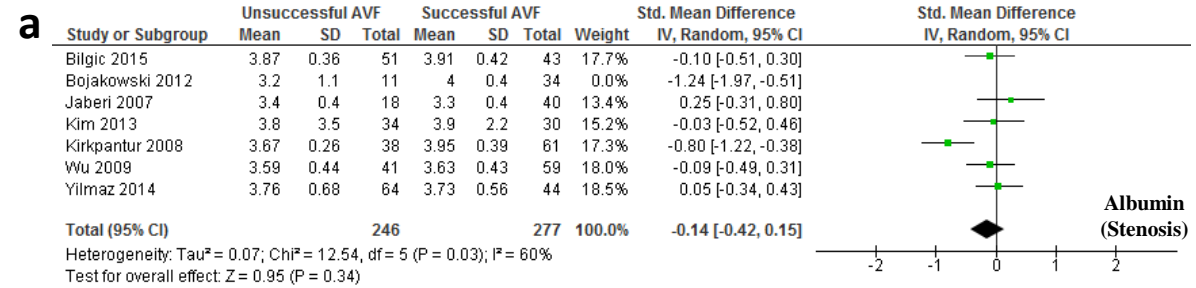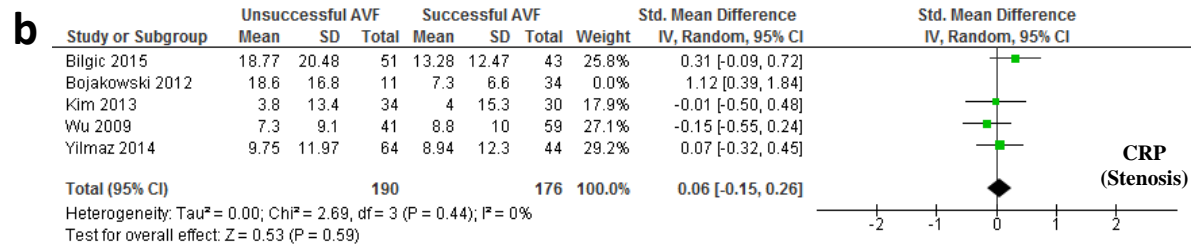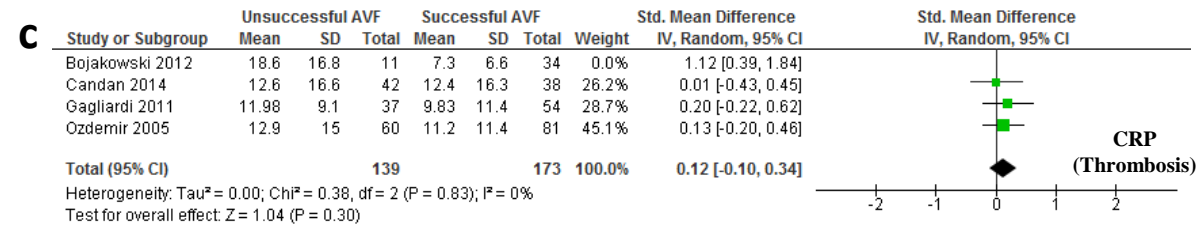

Supplement: S2 Fig — Forest plot of meta-analysis data showing the lack of an association between circulating albumin with AVF (a) stenosis; and circulating CRP with AVF (b) stenosis or (c) thrombosis, when data from Bojakowski et al. is removed. (PDF) [file pone.0159963.s002.pdf]
