# Supplementary material for: A Systematic Review and Meta-Analysis of Circulating Biomarkers Associated with Failure of Arteriovenous Fistulae for Haemodialysis
Source: PLoS One. 2016 Jul 26;11(7):e0159963. doi: 10.1371/journal.pone.0159963 (PMC4961283; doi:10.1371/journal.pone.0159963)
Supplement: S1 File — (PDF) [file pone.0159963.s003.pdf]

S1 File. Search Strategies.

All fields:	"AVF" AND "Vascular Access"
Title/Abstract:<br><br>*One term per search*	"biomarker" or "concentration" or "function" or "dysfunction" or "maturation" or "patency" or "failure" or "survival" or "thrombo*" or "steno*" or "factor" or "predict*" or "serum" or "plasma" or "circulating" or "risk factor" or "blood"

MEDLINE

Date limiter: January 1966 – December 2015
Date of Search: 22nd December 2015
Total Number of hits: 2266

EMBASE

Date limiter: January 1966 – December 2015
Date of Search: 22nd December 2015
Total Number of hits: 1817

COCHRANE LIBRARY

Date limiter: January 1966 – December 2015
Date of Search: 22nd December 2015
Total Number of hits: 152
