# Supplementary material for: A Systematic Review and Meta-Analysis of Circulating Biomarkers Associated with Failure of Arteriovenous Fistulae for Haemodialysis
Source: PLoS One. 2016 Jul 26;11(7):e0159963. doi: 10.1371/journal.pone.0159963 (PMC4961283; doi:10.1371/journal.pone.0159963)
Supplement: S2 File — (PDF) [file pone.0159963.s004.pdf]

Data Extraction Form

Reviewer:

First author:

Year of publication:

1. Study Design

What is the study design?

- ☐ Case control
- ☐ Cohort
  - ☐ Prospective
  - ☐ Retrospective
- ☐ Other: ____

Is the study single-centred or multi-centred?

- ☐ Single-centred
- ☐ Multi-centred

What is the total number of participants?

N = _______________ participants

Are the methods for recruitment and sampling clearly stated?

- ☐ Yes
- ☐ No

If so, how were patients recruited?

____

____

Was ethical approval stated? ____________

What were the inclusion and exclusion criteria for participants? (AVF patients)

Inclusion criteria: ____

____

____

Exclusion criteria:____

____

____

What was the AV fistula outcome being assessed?

- ☐ AV fistula failure
- ☐ Primary AV fistula failure
- ☐ AV fistula dysfunction
- ☐ AV fistula thrombosis
- ☐ Other: ____

Was a definition of a working provided?

- ☐ No
- ☐ Yes
  State: ____

How was this outcome diagnosed? (can tick more than one box)

- ☐ Clinical signs (i.e. inspection, palpation, auscultation)
- ☐ Dialysis failure
- ☐ Ultrasonography
- ☐ Angiogram/ fistulogram
- ☐ Unclear
- ☐ Other: ____

State the timing of blood collection:

- ☐ At AV fistula construction (i.e. peri-operative)
- ☐ At AV fistula failure
- ☐ At specified follow-up time. State _________________
- ☐ Unclear
- ☐ Other. State____

In brief, state the laboratory methods for quantifying biomarkers:

____

____

2a. Case control analysis

State the number of cases and controls.

Cases = _____________ Definition:____________________________________

Controls = ___________
Definition:____________________________________

Is the case group further divided into subgroups?

- ☐ Yes, state subgroups and number: _________________________________
- ☐ No

Were cases and controls matched for confounding factors?

- ☐ Yes,
- ☐ No
- ☐ Prospective study (cases & controls derived from common baseline population)

If so, list matched confounding factors (e.g. age, gender): ______________________

____

2b. Cohort studies

At baseline, did patients have any history of vascular access failure?

- ☐ Yes, describe: ____
- ☐ No, newly constructed fistula
- ☐ Unclear

What was the number of patients recruited? ________________________

What was the follow up time? ____________________________________

How many patients were lost to follow up? _________________________

What was the final number of participants analysed?

Cases: _________ Controls: __________

4. Statistical Analyses

What statistical analyses were employed?

- ☐ Comparative analyses
  - ☐ Qualitative
    - ☐ χ^2 (chi squared) test
    - ☐ Fisher's
  - ☐ Quantitative
    - ☐ Mann-Whitney U (non-parametric)
    - ☐ Student's t test (parametric)
  - ☐ Odds ratio
- ☐ Predictive analyses
  - ☐ Hazards ratio
  - ☐ Kaplan-Meier survival curve
  - ☐ Log rank test
- ☐ Other:____

____

Was the statistical significance set at $p < 0.05$?

- ☐ Yes
- ☐ No;

  State cut-off: ____

  Give reasons:

  ____

Were multivariate analyses performed?

- ☐ No
- ☐ Yes

  State any confounding factors that have been adjusted for:

3. Population Characteristics

State available population characteristics data for cases and controls (preferred) OR combined population. List any additional important characteristic data in blank rows.

**Item**	**Units**	**Cases**	**Controls**	**Combined Population**
% Males				
Age				
Smoking				
Hypertension				
Hypertension				
Dyslipidaemia				
BMI				
Radiocephalic: brachiocephalic				
Kt/v				
Duration of CRF				
Duration of HD				
Previous vascular access failure				

BMI, body mass index; CRF, chronic renal failure; HD, haemodialysis

State available medication data for cases and controls (preferred) OR combined population.

Medications	Dose	Cases	Controls	Combined Population
Anticoagulants				
Lipid altering medications				
Anti-hypertensives				

Oral hypoglycaemics				
Anti-inflammatory agents				
Other: __________				

4. Biomarkers Assessed

List all biomarkers that were assessed in relation to AV fistula outcome, and state the statistical analysis applied (e.g. survival curve, multivariate HR, univariate HR, OR, raw conc.)

**Biomarker**	**Statistical model(s) applied**

5. Results

Record details for survival curves/ log rank test:

**Biomarker**	**Units**	**Cut-off 1**	**Cut-off 2**	**Cut-off 3**	**Cut-off 4**	**Cut-off 5**	**P value** (log rank)

Record any hazards ratios, risk ratios or odds ratios stated for each biomarker. Record with the ratio is univariate or multivariate analysis

**Biomarker**	**OR/HR/RR**	**95 % CI**	**P value**	**Univariate/ multivariate**

More?	□ (record on additional data extraction forms)			

Record biomarker concentrations in case and control groups:

**Biomarker**	**Units**	**Cases**	**Controls**	**P value**

Record any additional results that support the association between circulating biomarker levels and AV fistula dysfunction.

____
____
____
____
____
____
____
____
____
____
____
____

7. Discussion and Conclusion

What conclusions were drawn from this study?

____
____
____
____
____

List limitations of the study identified by the author:

____
____
____
____
____

List any other limitations not stated by the author:
