# Supplementary material for: A Systematic Review and Meta-Analysis of Circulating Biomarkers Associated with Failure of Arteriovenous Fistulae for Haemodialysis
Source: PLoS One. 2016 Jul 26;11(7):e0159963. doi: 10.1371/journal.pone.0159963 (PMC4961283; doi:10.1371/journal.pone.0159963)
Supplement: S3 File — (PDF) [file pone.0159963.s005.pdf]

S3 File. Results of the modified Ottawa-Newcastle tool to assess risk of bias in cohort studies

Question	Reference								
	Baumann	Bojakowski	Gagliardi	Jaberi	Kaygin	Kirkpantur	Masaki	Wu	Yilmaz
1. Was selection of exposed and non-exposed cohorts drawn from the same population?	Definitely Yes	Probably Yes	Probably Yes	Probably Yes	Probably Yes	Definitely Yes	Probably Yes	Probably No	Probably No
2. Can we be confident in the assessment of exposure?	Definitely Yes	Definitely Yes	Definitely Yes	Probably Yes	Probably Yes	Probably Yes	Probably Yes	Probably No	Probably Yes
3. Can we be confident that the outcome of interest was not present at start of study?	Definitely Yes	Definitely Yes	Definitely Yes	Definitely No	Definitely Yes	Probably Yes	Probably Yes	Definitely No	Probably Yes
4. Did the study match exposed and unexposed for all variables that are associated with the outcome of interest, or did the statistical analysis adjust for these prognostic variables?	Mostly Yes	Mostly Yes	Mostly Yes	Mostly No	Mostly Yes	Mostly Yes	Mostly Yes	Mostly Yes	Mostly Yes
5. Can we be confident in the assessment of the presence or absence of prognostic factors?	Probably Yes	Definitely Yes	Definitely Yes	Probably Yes	Definitely Yes	Probably Yes	Definitely Yes	Probably Yes	Probably Yes
6. Can we be confident in the assessment of outcome?	Definitely Yes	Definitely Yes	Probably Yes	Probably Yes	Probably Yes	Definitely Yes	Definitely Yes	Definitely Yes	Definitely Yes
7. Was the follow up of cohorts adequate?	Definitely Yes	Definitely Yes	Probably Yes	Probably Yes	Definitely Yes	Probably Yes	Probably Yes	Probably Yes	Definitely Yes
8. Were co-interventions similar between groups?	Probably Yes	Definitely Yes	Probably Yes	Probably Yes	Probably Yes	Probably Yes	Probably Yes	Probably Yes	Probably Yes
Summary of overall risk of bias	Low	Very Low	Low	High	Low	Medium	Medium	High	High

NB: The risk of bias was considered very low if 100% questions were answered with either a 'Definitely Yes' or 'Probably/Mostly Yes', with at least 6/8 being 'Definitely Yes'; low if 100% questions were answered with either a 'Definitely Yes' or 'Probably/Mostly Yes', with at least 3/8 being 'Definitely Yes'; medium if 100% questions were answered with either a 'Definitely Yes' or 'Probably/Mostly Yes', with 2/8 or less being 'Definitely Yes'; High if 3/8 or less questions answered with either 'Definitely No' or 'Probably/Mostly No'; Very high if 4/8 or more questions were answered with either 'Definitely No' or 'Probably/Mostly No.'

Definitely Yes: Very low risk of bias; Probably/Mostly yes: Low risk of bias; Probably/Mostly no: Medium risk of bias; Definitely No: high risk of bias. Modified Ottawa-Newcastle Tool (https://distillercer.com/resources/)
