# Supplementary material for: A Systematic Review and Meta-Analysis of Circulating Biomarkers Associated with Failure of Arteriovenous Fistulae for Haemodialysis
Source: PLoS One. 2016 Jul 26;11(7):e0159963. doi: 10.1371/journal.pone.0159963 (PMC4961283; doi:10.1371/journal.pone.0159963)
Supplement: S4 File — (PDF) [file pone.0159963.s006.pdf]

S4 File. Results of the modified Ottawa-Newcastle tool to assess risk of bias in case control studies

Question	Reference			
	Bilgic	Candan	Kim	Ozdemir
1. Can we be confident in the assessment of exposure?	Probably Yes	Definitely Yes	Definitely Yes	Probably Yes
2. Can we be confident that cases had developed the outcome of interest and controls had not?	Definitely Yes	Probably Yes	Definitely Yes	Probably Yes
3. Were the cases (those who were exposed and developed the outcome of interest) properly selected?	Definitely Yes	Definitely Yes	Probably Yes	Probably Yes
4. Were the controls (those who were exposed and did not develop the outcome of interest) properly selected?	Definitely Yes	Definitely Yes	Probably Yes	Probably Yes
5. Were cases and controls matches according to important prognostic variables or was statistical adjustment carried out for those variables?	Probably Yes	Probably Yes	Probably Yes	Probably Yes
Summary of overall risk of bias	Low	Low	Medium	Medium

NB: The risk of bias was considered very low if 100% of questions were answered with ‘Definitely Yes’; low if 100% of questions were answered with either a ‘Definitely Yes’ or ‘Probably/Mostly Yes’, with at least 3/5 being ‘Definitely Yes’; Medium if 100% of questions were answered with either a ‘Definitely Yes’ or ‘Probably/Mostly Yes’, with at least 2/5 or less being ‘Definitely Yes’; High if 2/5 or less questions answered with either ‘Definitely No’ or ‘Probably/Mostly No’; Very high if 3/5 or more questions answered with either ‘Definitely No’ or ‘Probably/Mostly No.’

Definitely Yes: Very low risk of bias; Probably/Mostly yes: Low risk of bias; Probably/Mostly no: Medium risk of bias; Definitely No: high risk of bias.

Modified Ottawa-Newcastle Tool (https://distillercer.com/resources/)
