# Supplementary material for: A Systematic Review and Meta-Analysis of Circulating Biomarkers Associated with Failure of Arteriovenous Fistulae for Haemodialysis
Source: PLoS One. 2016 Jul 26;11(7):e0159963. doi: 10.1371/journal.pone.0159963 (PMC4961283; doi:10.1371/journal.pone.0159963)
Supplement: S5 File — (PDF) [file pone.0159963.s007.pdf]

S5 File. Leave-one-out sensitivity analyses for all meta-analyses

Leave-one-out sensitivity test for the meta-analysis assessing Albumin and all AVF failure

Study Excluded	No. Studies	N_{Cases}	$N_{Controls}$	SMD (95%CI)	*p* value*	I^2	I^2 *p* value
Nil	10	411	714	-0.44 [-0.95, 0.07]	0.09	93%	< **0.001**
Bilgic	9	360	671	-0.48 [-1.04, 0.08]	0.09	93%	< **0.001**
Bojakowski	9	400	680	-0.36 [-0.90, 0.18]	0.19	94%	< **0.001**
Candan	9	369	676	-0.49 [-1.04, 0.06]	0.08	93%	< **0.001**
Gagliardi	9	374	660	-0.43 [-1.00, 0.14]	0.14	94%	< **0.001**
Jaberi	9	393	674	-0.51 [-1.05, 0.02]	0.06	93%	< **0.001**
Kaygin	9	336	403	-0.25 [-0.51, 0.02]	0.07	66%	**<0.003**
Kim	9	377	684	-0.48 [-1.03, 0.07]	0.08	93%	< **0.001**
Kirkpantur	9	373	653	-0.04 [-0.97, 0.17]	0.17	94%	< **0.001**
Wu	9	370	655	-0.48 [-1.04, 0.08]	0.09	93%	< **0.001**
Yilmaz	9	347	670	-0.50 [-1.05, 0.05]	0.08	93%	< **0.001**

SMD: standardised mean difference; I^2: heterogeneity index; *Calculated according to inverse-variance random-effects model. P-values in bold considered significant.

Leave-one-out sensitivity test for the meta-analysis assessing Calcium and all AVF failure

Study Excluded	No. Studies	N_{Cases}	$N_{Controls}$	SMD (95%CI)	*p* value*	I^2	I^2 *p* value
Nil	5	208	216	-0.04 [-0.24, 0.15]	0.67	0%	0.86
Bilgic	4	157	173	-0.00 [-0.23, 0.22]	0.96	0%	0.85
Jaberi	4	190	176	-0.06 [-0.27, 0.14]	0.54	0%	0.82
Kim	4	174	186	-0.05 [-0.26, 0.17]	0.67	0%	0.73
Wu	4	167	157	-0.08 [-0.30, 0.14]	0.47	0%	0.86
Yilmaz	4	144	172	-0.01 [-0.24, 0.21]	0.91	0%	0.80

SMD: standardised mean difference; I^2: heterogeneity index; *Calculated according to inverse-variance random-effects model. P-values in bold considered significant.

Leave-one-out sensitivity test for the meta-analysis assessing Creatinine and all AVF failure

Study Excluded	No. Studies	N_{Cases}	$N_{Controls}$	SMD (95%CI)	*p* value*	I^2	I^2 *p* value
Nil	3	127	404	0.08 [-0.13, 0.28]	0.46	0%	0.84
Bojakowski	2	116	370	0.08 [-0.13, 0.29]	0.46	0%	0.56
Kaygin	2	52	93	0.15 [-0.20, 0.49]	0.40	0%	0.75
Wu	2	86	345	0.04 [-0.20, 0.28]	0.73	0%	0.97

SMD: standardised mean difference; I^2: heterogeneity index; *Calculated according to inverse-variance random-effects model. P-values in bold considered significant.

Leave-one-out sensitivity test for the meta-analysis assessing CRP and all AVF failure

Study Excluded	No. Studies	N_{Cases}	$N_{Controls}$	SMD (95%CI)	*p* value*	I^2	I^2 *p* value
Nil	9	415	694	0.75 [-0.32, 1.82]	0.17	98%	< **0.001**
Bilgic	8	364	651	0.81 [-0.41, 2.02]	0.19	98%	< **0.001**
Bojakowski	8	404	660	0.71 [-0.45, 1.86]	0.23	98%	< **0.001**
Candan	8	373	656	0.84 [-0.35, 2.04]	0.17	98%	< **0.001**
Gagliardi	8	378	640	0.82 [-0.39, 2.03]	0.18	98%	< **0.001**
Kaygin	8	340	383	0.14 [-0.05, 0.33]	0.14	35%	0.15
Kim	8	381	664	0.85 [-0.34, 2.03]	0.16	98%	< **0.001**
Ozdemir	8	355	613	0.83 [-0.41, 2.07]	0.19	98%	< **0.001**
Wu	8	374	635	0.87 [-0.33, 2.06]	0.16	98%	< **0.001**
Yilmaz	8	351	650	0.84 [-0.38, 2.05]	0.18	98%	< **0.001**

CRP: C-reactive protein; SMD: standardised mean difference; I^2: heterogeneity index; *Calculated according to inverse-variance random-effects model. P-values in bold considered significant.

Leave-one-out sensitivity test for the meta-analysis assessing Ferritin and all AVF failure

Study Excluded	No. Studies	N_{Cases}	$N_{Controls}$	SMD (95%CI)	*p* value*	I^2	I^2 *p* value
Nil	5	228	240	-0.01 [-0.19, 0.18]	0.92	0%	0.92
Bilgic	4	177	197	0.01 [-0.20, 0.21]	0.95	0%	0.85
Bojakowski	4	217	206	0.01 [-0.18, 0.20]	0.93	0%	0.93
Candan	4	186	202	0.00 [-0.20, 0.20]	1.00	0%	0.84
Ozdemir	4	168	159	-0.05 [-0.28, 0.17]	0.63	0%	0.95
Yilmaz	4	164	196	-0.02 [-0.23, 0.20]	0.88	0%	0.83

SMD: standardised mean difference; I^2: heterogeneity index; *Calculated according to inverse-variance random-effects model. P-values in bold considered significant.

Leave-one-out sensitivity test for the meta-analysis assessing Haemoglobin and all AVF failure

Study Excluded	No. Studies	N_{Cases}	$N_{Controls}$	SMD (95%CI)	*p* value*	I^2	I^2 *p* value
Nil	6	224	260	-0.10 [-0.33, 0.14]	0.42	36%	0.17
Bilgic	5	173	217	-0.12 [-0.42, 0.18]	0.43	48%	0.10
Bojakowski	5	213	226	-0.02 [-0.22, 0.17]	0.80	0%	0.57
Candan	5	182	222	-0.16 [-0.41, 0.09]	0.21	30%	0.22
Jaberi	5	206	220	-0.12 [-0.40, 0.16]	0.40	48%	0.10
Kirkpantur	5	224	260	-0.10 [-0.33, 0.14]	0.42	38%	0.17
Yilmaz	5	160	216	-0.14 [-0.43, 0.15]	0.35	45%	0.12

SMD: standardised mean difference; I^2: heterogeneity index; *Calculated according to inverse-variance random-effects model. P-values in bold considered significant.

Leave-one-out sensitivity test for the meta-analysis assessing HDL-C and all AVF failure

Study Excluded	No. Studies	N_{Cases}	$N_{Controls}$	SMD (95%CI)	*p* value*	I^2	I^2 *p* value
Nil	7	322	590	-0.45 [-1.12, 0.23]	0.20	95%	< **0.001**
Bilgic	6	271	547	-0.54 [-1.33, 0.26]	0.19	95%	< **0.001**
Bojakowski	6	311	556	-0.48 [-1.23, 0.28]	0.22	96%	< **0.001**
Candan	6	280	552	-0.54 [-1.33, 0.24]	0.17	95%	< **0.001**
Kaygin	6	247	279	-0.57 [-1.37, 0.22]	0.16	64%	< **0.001**
Kirkpantur	6	284	529	-0.18 [-0.73, 0.37]	0.52	91%	< **0.001**
Wu	6	281	531	-0.56 [-1.35, 0.23]	0.16	95%	< **0.001**
Yilmaz	6	258	546	-0.25 [-0.88, 0.38]	0.44	93%	< **0.001**

HDL-C: high density lipoprotein cholesterol; SMD: standardised mean difference; I^2: heterogeneity index; *Calculated according to inverse-variance random-effects model. P-values in bold considered significant.

Leave-one-out sensitivity test for the meta-analysis assessing LDL-C and all AVF failure

Study Excluded	No. Studies	N_{Cases}	$N_{Controls}$	SMD (95%CI)	*p* value*	I^2	I^2 *p* value
Nil	7	322	590	-0.06 [-0.64, 0.53]	0.85	93%	< **0.001**
Bilgic	6	271	547	-0.21 [-0.83, 0.42]	0.52	93%	< **0.001**
Bojakowski	6	311	556	-0.09 [-0.74, 0.55]	0.77	94%	< **0.001**
Candan	6	280	552	-0.08 [-0.76, 0.61]	0.82	94%	< **0.001**
Kaygin	6	247	279	-0.09 [-0.86, 0.67]	0.81	94%	< **0.001**
Kirkpantur	6	284	529	0.27 [0.04, 0.50]	**0.02**	49%	0.08
Wu	6	281	531	-0.13 [-0.81, 0.56]	0.72	94%	< **0.001**
Yilmaz	6	258	546	-0.08 [-0.78, 0.62]	0.82	94%	< **0.001**

LDL-C: low density lipoprotein cholesterol; SMD: standardised mean difference; I^2: heterogeneity index; *Calculated according to inverse-variance random-effects model. P-values in bold considered significant.

Leave-one-out sensitivity test for the meta-analysis assessing PTH and all AVF failure

Study Excluded	No. Studies	N_{Cases}	$N_{Controls}$	SMD (95%CI)	*p* value*	I^2	I^2 *p* value
Nil	4	217	206	-0.04 [-0.23, 0.15]	0.67	0%	0.83
Bilgic	3	166	163	-0.08 [-0.29, 0.14]	0.50	0%	0.79
Candan	3	175	168	-0.01 [-0.23, 0.20]	0.90	0%	0.76
Ozdemir	3	157	125	-0.01 [-0.24, 0.23]	0.94	0%	0.72
Yilmaz	3	153	162	-0.07 [-0.29, 0.16]	0.56	0%	0.71

PTH: parathyroid hormone; SMD: standardised mean difference; I^2: heterogeneity index; *Calculated according to inverse-variance random-effects model. P-values in bold considered significant.

Leave-one-out sensitivity test for the meta-analysis assessing Phosphorus and all AVF failure

Study Excluded	No. Studies	N_{Cases}	$N_{Controls}$	SMD (95%CI)	*p* value*	I^2	I^2 *p* value
Nil	5	208	216	0.10 [-0.10, 0.30]	0.32	4%	0.39
Bilgic	4	157	173	0.13 [-0.13, 0.39]	0.32	25%	0.26
Jaberi	4	190	176	0.03 [-0.18, 0.24]	0.79	0%	0.97
Kim	4	174	186	0.13 [-0.12, 0.38]	0.31	25%	0.26
Wu	4	167	157	0.15 [-0.10, 0.39]	0.23	16%	0.31
Yilmaz	4	144	172	0.11 [-0.16, 0.38]	0.42	28%	0.25

SMD: standardised mean difference; I^2: heterogeneity index; *Calculated according to inverse-variance random-effects model. P-values in bold considered significant.

Leave-one-out sensitivity test for the meta-analysis assessing Total Cholesterol and all AVF failure

Study Excluded	No. Studies	N_{Cases}	$N_{Controls}$	SMD (95%CI)	*p* value*	I^2	I^2 *p* value
Nil	4	192	464	0.14 [-0.12, 0.41]	0.28	50%	0.11
Candan	3	150	426	0.14 [-0.22, 0.50]	0.45	67%	**0.05**
Gagliardi	3	155	410	0.07 [-0.24, 0.39]	0.67	58%	0.09
Kaygin	3	117	153	0.08 [-0.30, 0.47]	0.67	59%	0.08
Kirkpantur	3	154	403	0.27 [0.07, 0.46]	**0.007**	0%	0.73

SMD: standardised mean difference; I^2: heterogeneity index; *Calculated according to inverse-variance random-effects model. P-values in bold considered significant.

Leave-one-out sensitivity test for the meta-analysis assessing Triglycerides and all AVF failure

Study Excluded	No. Studies	N_{Cases}	$N_{Controls}$	SMD (95%CI)	*p* value*	I^2	I^2 *p* value
Nil	7	322	590	-0.02 [-0.17, 0.12]	0.74	0%	0.70
Bilgic	6	271	547	-0.01 [-0.17, 0.14]	0.86	0%	0.60
Bojakowski	6	311	556	-0.03 [-0.18, 0.12]	0.68	0%	0.61
Candan	6	280	552	-0.04 [-0.20, 0.11]	0.58	0%	0.67
Kaygin	6	247	279	-0.00 [-0.18, 0.17]	0.96	0%	0.60
Kirkpantur	6	284	529	0.01 [-0.14, 0.17]	0.88	0%	0.84
Wu	6	281	531	-0.06 [-0.21, 0.09]	0.44	0%	0.82
Yilmaz	6	258	546	-0.02 [-0.18, 0.13]	0.78	0%	0.58

SMD: standardised mean difference; I^2: heterogeneity index; *Calculated according to inverse-variance random-effects model. P-values in bold considered significant.

Leave-one-out sensitivity test for the meta-analysis assessing Albumin and AVF stenosis

Study Excluded	No. Studies	N_{Cases}	$N_{Controls}$	SMD (95%CI)	*p* value*	I^2	I^2 *p* value
Nil	7	257	311	-0.24 [-0.57, 0.08]	0.14	71%	**0.002**
Bilgic	6	206	268	-0.28 [-0.67, 0.11]	0.17	75%	**0.001**
Bojakowski	6	246	277	-0.14 [-0.42, 0.15]	0.34	60%	**0.03**
Jaberi	6	239	271	-0.32 [-0.66, 0.03]	0.07	72%	**0.003**
Kim	6	223	281	-0.28 [-0.66, 0.09]	0.14	75%	**0.001**
Kirkpantur	6	219	250	-0.13 [-0.42, 0.16]	0.39	56%	**0.04**
Wu	6	216	252	-0.28 [-0.67, 0.11]	0.16	75%	**0.001**
Yilmaz	6	193	267	-0.30 [-0.68, 0.07]	0.12	73%	**0.002**
Nil †	6	246	277	-0.14 [-0.42, 0.15]	0.34	60%	**0.03**
Bilgic †	5	195	234	-0.14 [-0.49, 0.21]	0.43	68%	**0.01**
Jaberi †	5	228	237	-0.20 [-0.50, 0.11]	0.20	62%	**0.03**
Kim †	5	212	247	-0.15 [-0.49, 0.18]	0.37	67%	**0.02**
Kirkpantur †	5	208	216	-0.01 [-0.20, 0.19]	0.93	0%	0.87
Wu †	5	205	218	-0.14 [-0.49, 0.21]	0.42	68%	**0.01**
Yilmaz †	5	182	233	-0.18 [-0.51, 0.16]	0.31	65%	**0.02**

SMD: standardised mean difference; I^2: heterogeneity index; *Calculated according to inverse-variance random-effects model. P-values in bold considered significant. †Analysis excluded mixed population data from Bojakowski *et al*.

Leave-one-out sensitivity test for the meta-analysis assessing Albumin and AVF thrombosis

Study Excluded	No. Studies	N_{Cases}	$N_{Controls}$	SMD (95%CI)	*p* value*	I^2	I^2 *p* value
Nil	3	90	126	-0.53 [-1.14, 0.08]	0.09	77%	**0.01**
Bojakowski	2	79	92	-0.26 [-0.78, 0.25]	0.31	64%	0.09
Candan	2	48	88	-0.81 [-1.50, -0.13]	**0.02**	64%	0.10
Gagliardi	2	53	72	-0.58 [-1.79, 0.63]	0.35	88%	**0.004**

SMD: standardised mean difference; I^2: heterogeneity index; *Calculated according to inverse-variance random-effects model. P-values in bold considered significant.

Leave-one-out sensitivity test for the meta-analysis assessing CRP and AVF stenosis

Study Excluded	No. Studies	N_{Cases}	$N_{Controls}$	SMD (95%CI)	*p* value*	I^2	I^2 *p* value
Nil	5	201	210	0.19 [-0.14, 0.52]	0.26	61%	**0.04**
Bilgic	4	150	167	0.17 [-0.25, 0.59]	0.42	68%	**0.02**
Bojakowski	4	190	176	0.06 [-0.15, 0.26]	0.59	0%	0.44
Kim	4	167	180	0.26 [-0.16, 0.67]	0.23	70%	**0.02**
Wu	4	160	151	0.29 [-0.09, 0.68]	0.13	61%	0.06
Yilmaz	4	137	166	0.25 [-0.20, 0.69]	0.27	70%	**0.02**
Nil †	4	190	176	0.06 [-0.15, 0.26]	0.59	0%	0.44
Bilgic †	3	139	133	-0.03 [-0.27, 0.21]	0.79	0%	0.73
Kim †	3	156	146	0.07 [-0.19, 0.33]	0.59	23%	0.27
Wu †	3	149	117	0.13 [-0.11, 0.38]	0.28	0%	0.54
Yilmaz †	3	126	132	0.05 [-0.24,0.34]	0.72	26%	0.26

CRP: C-reactive protein; SMD: standardised mean difference; I^2: heterogeneity index; *Calculated according to inverse-variance random-effects model. P-values in bold considered significant. †Analysis excluded mixed population data from Bojakowski *et al*.

Leave-one-out sensitivity test for the meta-analysis assessing CRP and AVF thrombosis

**Study Excluded**	**No. Studies**	**N_{Cases}**	**$N_{Controls}$**	**SMD (95%CI)**	***p* value***	**I^2**	**I^2 *p* value**
Nil	4	150	207	0.27 [-0.08, 0.62]	0.13	57%	0.07
Bojakowski	3	139	173	0.12 [-0.10, 0.34]	0.30	0%	0.83
Candan	3	108	169	0.38 [-0.08, 0.85]	0.11	67%	**0.05**
Gagliardi	3	113	153	0.33 [-0.18, 0.84]	0.21	72%	**0.03**
Ozdemir	3	90	126	0.37 [-0.17, 0.91]	0.18	70%	**0.04**
Nil †	3	139	173	0.12 [-0.10, 0.34]	0.30	0%	0.83
Candan †	2	97	135	0.16 [-0.10, 0.42]	0.24	0%	0.79
Gagliardi †	2	102	119	0.09 [-0.18, 0.35]	0.52	0%	0.68
Ozdemir †	2	79	92	0.11 [-0.19, 0.41]	0.47	0%	0.54

CRP: C-reactive protein; SMD: standardised mean difference; I^2: heterogeneity index; *Calculated according to inverse-variance random-effects model. P-values in bold considered significant.
†Analysis excluded mixed population data from Bojakowski *et al*.
