# Supplementary material for: A Systematic Review and Meta-Analysis of Circulating Biomarkers Associated with Failure of Arteriovenous Fistulae for Haemodialysis
Source: PLoS One. 2016 Jul 26;11(7):e0159963. doi: 10.1371/journal.pone.0159963 (PMC4961283; doi:10.1371/journal.pone.0159963)
Supplement: S1 Table — PTH: parathyroid hormone; HDL-C: high density lipoprotein cholesterol; LDL-C: low density lipoprotein cholesterol; CRP: C-reactive protein; sE-Selectin: soluble E-selectin; eEPCR: soluble endothelial protein C receptor; TG: triglycerides; WBC: white blood cell count; MCV: mean corpuscular volume; MCH: mean corpuscular haemoglobin; RDW: red blood cell distribution width; RBC: red blood cell count; Ca x P: calcium times phosphate; VEGF-A: vascular endothelial growth factor A; MIS: malnutrition inflammation score; CMV: cytomegalovirus; OPG: osteoprotegerin; TC: total cholesterol; ADMA: asymmetrical dimethylarginine; NLR: neutrophil-lymphocyte ratio. (DOCX) [file pone.0159963.s008.docx]

| **S1 Table. Blood collection and laboratory methods used to quantify biomarkers** | | | | |
| --- | --- | --- | --- | --- |
| **Reference** | **Blood Collection** | **Biomarkers** | **Quantification Method** | **Blood medium** |
| **Baumann [8]** | Pre-operative | Fibrinogen | Automated analyser | n/r |
| **Bilgic [9]** | At Failure | Albumin | n/r | n/r |
|  |  | Calcium | Automated analyser | Serum |
|  |  | Phosphorus | Automated analyser | Serum |
|  |  | PTH | n/r | n/r |
|  |  | HDL-C | Automated analyser | Serum |
|  |  | TG | Automated analyser | Serum |
|  |  | LDL-C | Automated analyser | Serum |
|  |  | Haemoglobin | n/r | n/r |
|  |  | Transferrin Saturation | n/r | n/r |
|  |  | Ferritin | n/r | n/r |
|  |  | CRP | Automated analyser | Serum |
|  |  | sE-Selectin | ELISA | Serum |
|  |  | sEPCR | ELISA | Plasma |
| **Bojakowski [10]** | Peri-operative | CRP | Automated analyser | n/r |
|  |  | Procalcitonin | Automated analyser | n/r |
|  |  | HDL-C | Automated analyser | n/r |
|  |  | LDL-C | Automated analyser | n/r |
|  |  | TG | Automated analyser | n/r |
|  |  | Creatinine | Automated analyser | n/r |
|  |  | Albumin | Automated analyser | Serum |
|  |  | Albumin corrected Calcium | Automated analyser | Serum |
|  |  | WBC | Automated analyser | n/r |
|  |  | Neutrophils | Automated analyser | n/r |
|  |  | Lymphocytes | Automated analyser | n/r |
|  |  | Monocytes | Automated analyser | n/r |
|  |  | Hematocrit | Automated analyser | n/r |
|  |  | MCV | Automated analyser | n/r |
|  |  | MCH | Automated analyser | n/r |
|  |  | RDW | Automated analyser | n/r |
|  |  | Platelets | Automated analyser | n/r |
|  |  | Iron | Automated analyser | n/r |
|  |  | Transferrin | Automated analyser | n/r |
|  |  | Ferritin | Automated analyser | n/r |
|  |  | RBC | Automated analyser | n/r |
| **Candan [11]** | Functional fasted mid-week HD session | Ca x P | n/r | n/r |
|  |  | PTH | n/r | n/r |
|  |  | Haemoglobin | n/r | n/r |
|  |  | WBC | n/r | n/r |
|  |  | Platelets | n/r | n/r |
|  |  | Total cholesterol | n/r | n/r |
|  |  | TG | n/r | n/r |
|  |  | LDL-C | n/r | n/r |
|  |  | HDL-C | n/r | n/r |
|  |  | Albumin | n/r | n/r |
|  |  | CRP | n/r | n/r |
|  |  | Ferritin | n/r | n/r |
|  |  | Transferrin Saturation | n/r | n/r |
|  |  | VEGF-A | ELISA | Plasma |
| **Gagliardi [12]** | Mean of annual blood results | TC | Col-esterase + oxidase POD method | Plasma |
|  |  | Fibrinogen | Clauss method | Plasma |
|  |  | CRP | Automated analyser | n/r |
|  |  | MIS | n/r | n/4 |
|  |  | Albumin | n/r | Serum |
|  |  | Anti-CMV IgG | Chemiluminescent immunoassay | Serum |
|  |  | Anti-H. *pylori* IgG | ELISA | Serum |
|  |  | Anti-C. *pneumoniae* IgG | Micro-immunofluorescent assay | Serum |
| **Jaberi [13]** | Unclear | Haemoglobin | Automated analyser | n/r |
|  |  | Neutrophils | Automated analyser | n/r |
|  |  | Albumin | Dichromatic digital endpoint method | n/r |
|  |  | Calcium | Indirect potentiametry | n/r |
|  |  | Phosphorus | A time-rated method | n/r |
|  |  | Platelets | Automated analyser | n/r |
|  |  | PTH | n/r | n/r |
|  |  | Ferritin | n/r | n/r |
|  |  | Ca-PO_4_ | n/r | n/r |
| **Kaygin [14]** | Peri-operative | TC | n/r | Serum |
|  |  | HDL-C | n/r | Serum |
|  |  | LDL-C | n/r | Serum |
|  |  | TG | n/r | Serum |
|  |  | Albumin | Automated analyser | Serum |
|  |  | CRP | Nephelometry | Serum |
|  |  | Fibrinogen | Automated analyser | Serum |
| **Kim [15]** | Mean over 6 months | Fetuin-A | ELISA | n/r |
|  |  | OPG | ELISA | Serum |
|  |  | Heatshock protein 70 | ELISA | Serum |
|  |  | Uric Acid | n/r | n/r |
|  |  | Calcium | n/r | n/r |
|  |  | Phosphorus | n/r | n/r |
|  |  | Ca x P | n/r | n/r |
|  |  | PTH | Immunoradiometric assay | n/r |
|  |  | TG | Automated analyser | Serum |
|  |  | TC | Automated analyser | Serum |
|  |  | LDL-C | Automated analyser | Serum |
|  |  | HDL-C | Automated analyser | Serum |
|  |  | CRP | Automated analyser | Serum |
|  |  | Albumin | Automated analyser | Serum |
| **Kirkpantur [16]** | Peri-operative | Haemoglobin | Ultraviolet assay | n/r |
|  |  | Albumin | Ultraviolet assay | Serum |
|  |  | CRP | Nephelometry | Serum |
|  |  | TC | Ultraviolet assay | Serum+Plasma |
|  |  | TG | Ultraviolet assay | Serum |
|  |  | LDL-C | Friedewald formula | Serum |
|  |  | HDL-C | Precipitation | Serum+Plasma |
|  |  | Glucose | Glucose-oxidase method | Plasma |
| **Masaki [17]** | At failure | Hematocrit | n/r | n/r |
| **Ozdemir [18]** | 6 months prior AVF failure | Hamatocrit | Automated analyser | n/r |
|  |  | Eosinophil | Automated analyser | Whole blood |
|  |  | Ferritin | Automated analyser | Serum |
|  |  | CRP | Latex agglutination | Serum |
|  |  | PTH | Automated analyser | Serum |
| **Wu [19]** | Peri-operative | LDL-C | Automated analyser | Plasma |
|  |  | HDL-C | Automated analyser | Plasma |
|  |  | TG | Automated analyser | Plasma |
|  |  | Calcium | Automated analyser | Plasma |
|  |  | Phosphorus | Automated analyser | Plasma |
|  |  | Albumin | Automated analyser | Plasma |
|  |  | Creatinine | Automated analyser | Plasma |
|  |  | CRP | Automated analyser | Plasma |
|  |  | Homocysteine | ELISA | Plasma |
|  |  | ADMA | ELISA | Plasma |
| **Yilmaz [20]** | 6 months prior to failure | Albumin | n/r | Serum |
|  |  | Calcium | n/r | Serum |
|  |  | Phosphorus | n/r | Serum |
|  |  | Ca x P | n/r | Serum |
|  |  | PTH | n/r | n/r |
|  |  | HDL-C | n/r | Serum |
|  |  | LDL-C | n/r | Serum |
|  |  | Haemoglobin | n/r | Serum |
|  |  | Transferrin saturation | n/r | n/r |
|  |  | Ferritin | n/r | n/r |
|  |  | CRP | n/r | n/r |
|  |  | Uric acid | n/r | n/r |
|  |  | WBC | n/r | n/r |
|  |  | NLR | n/r | n/r |

PTH: parathyroid hormone; HDL-C: high density lipoprotein cholesterol; LDL-C: low density lipoprotein cholesterol; CRP: C-reactive protein; sE-Selectin: soluble E-selectin; eEPCR: soluble endothelial protein C receptor; TG: triglycerides; WBC: white blood cell count; MCV: mean corpuscular volume; MCH: mean corpuscular haemoglobin; RDW: red blood cell distribution width; RBC: red blood cell count; Ca x P: calcium times phosphate; VEGF-A: vascular endothelial growth factor A; MIS: malnutrition inflammation score; CMV: cytomegalovirus; OPG: osteoprotegerin; TC: total cholesterol; ADMA: asymmetrical dimethylarginine; NLR: neutrophil-lymphocyte ratio.
